# Global research trends and hotspots of colorectal cancer organoids: a bibliometric insight and visualization analysis via multiple databases

**DOI:** 10.3389/fonc.2026.1827951

**Published:** 2026-05-18

**Authors:** Xiaoyi Zhang, Xingyu Zhu, Yibing Chen, Bingye Lu

**Affiliations:** 1 Clinical Medical College, Chengdu University of Traditional Chinese Medicine, Chengdu, China; 2 The First Clinical Medical College, Nanjing Medical University, Nanjing, China

**Keywords:** bibliometric analysis, colorectal cancer, hotspots, organoid, personalized medicine, research trends, tumor microenvironment

## Abstract

**Objectives:**

Bibliometrics was adopted to analyze the research trends of colorectal cancer organoids, and to identify the global research hotspots and prospective directions.

**Methods:**

Publications related to colorectal cancer organoids published from January 1, 2010 to December 31, 2025 were obtained from the Web of Science Core Collection (WOSCC) and Scopus databases. Bibliometric and visualization analyses were conducted utilizing Bibliometrix R package, Python, CiteSpace and VOSviewer to explore the publication trends, countries, institutions, journals, authors and thematic evolution.

**Results:**

A total of 2239 relevant publications were included. The analysis revealed an explosive growth in the number of publications in this field over the past 16 years. China and the United States were the main contributors, and Utrecht University was the most productive institution. Hans Clevers had the highest total citations and number of publications, reflecting his leading influence in the field. *Cancers* was the most productive journal, while *Nature* was the most frequently cited. Keyword analysis and thematic evolution revealed that the current research hotspots primarily include drug screening, the tumor microenvironment, tumor immunity, and precision oncology.

**Conclusion:**

Research on colorectal cancer organoids is shifting from the construction and optimization of conventional organoid models to the development of high-fidelity disease models. Current studies focus on the simulation of the tumor microenvironment and personalized cancer therapy, and colorectal cancer organoids will achieve broader clinical applications in precision oncology in the future.

## 1 Introduction

Colorectal cancer (CRC) is the third most prevalent malignancy and the second most common cause of cancer mortality worldwide (1). While colorectal cancer incidence is declining among older adults in high-income countries, rates continue to rise in emerging economies and among young adults (age <50 years) worldwide (2, 3). Distant organ metastasis is the leading cause of death from CRC, with a 5-year relative survival rate of only approximately 14% (4). Surgery is the main treatment for CRC, supplemented by adjuvant chemotherapy and radiotherapy (5). However, treatment is often hindered by drug resistance (6). Additionally, many patients are diagnosed at advanced stages, losing the opportunity for curative surgery, or suffer from postoperative recurrence and metastasis. To improve both basic research and clinical management of CRC, traditional preclinical models are no longer adequate to meet current demands, necessitating the exploration of preclinical models with high fidelity (7).

Organoids are three-dimensional *in vitro* cultured tissues that recapitulate key structural and functional aspects of their *in vivo* counterparts. They can be derived from pluripotent stem cells, tissue-resident stem cells, progenitor cells or differentiated cells from normal or diseased tissues (e.g., epithelial and non-epithelial tumors) (8, 9). Patient-derived tumor organoids (PDTOs) can maintain interpatient heterogeneity, accurately recapitulate the histological and molecular characteristics of original tumors, and enable long-term culture with structural and genetic integrity (10–12). Currently, an increasing number of prospective clinical trials employ patient-derived tumor organoids (PDTOs) to evaluate drug responses and guide cancer treatment, particularly for colorectal tumors (13). Colorectal cancer organoids are also being employed to investigate cancer differentiation, invasion and metastasis, and hold promising potential for applications in personalized medicine (14–18).

Since 2010, the number of publications on colorectal cancer organoids has grown explosively. However, systematic quantitative analyses of the academic structure, research trends and emerging frontiers in this field are still lacking. In contrast to traditional narrative reviews, bibliometrics employs statistical approaches for the quantitative analysis of scientific literature, serving as a robust tool to reveal the knowledge structure within databases (19, 20). Such analysis not only reveals the fundamental status and research hotspots of the field, but also provides valuable insights and guidance for future studies focusing on model optimization, precision medicine, and interdisciplinary translational research. Therefore, this study utilized bibliometric methods to quantitatively and visually clarify its academic evolution and provide valuable insights into future developmental trends.

In recent years, driven by the rapid advancement of organoid research, related bibliometric and knowledge mapping analyses have increasingly emerged (21–25). Existing studies have largely provided macroscopic overviews of the organoid field or encompassed various disease types, offering valuable references for understanding its overall development. However, systematic analyses focusing on specific tumor types remain relatively insufficient within this body of literature, with studies specifically dedicated to CRC organoids being particularly limited.

Against this backdrop, the present study focuses specifically on CRC organoids. Utilizing a larger, more up-to-date dataset spanning an extended timeframe, we combine multiple bibliometric and visual analysis methods to systematically delineate research hotspots and developmental trends. Furthermore, we place particular emphasis on translational directions such as drug screening, the tumor microenvironment, and personalized medicine, thereby providing a more disease-specific and clinically-oriented analytical perspective.

## 2 Materials and methods

### 2.1 Data sources and processing

The literature search was implemented on February 09, 2026, utilizing the Web of Science Core Collection (WOSCC) database and Scopus database. The Scopus CSV files were downloaded and converted into plain text format by Python (version 3.14) to unify the data.

To realize data uniformity, this study adopted Python (version 3.14) to translate Scopus CSV files into a plain text format compatible with WOSCC. We also utilized Bibliometrix R package (v5.2.1) to perform data cleaning steps, including DOI-based deduplication, removal of records with “[Anonymous]” in the author field, and merging and standardization of duplicate institution names. Furthermore, the disambiguation function of the Bibliometrix R package was utilized.

### 2.2 Search strategy

**Figure 1:**
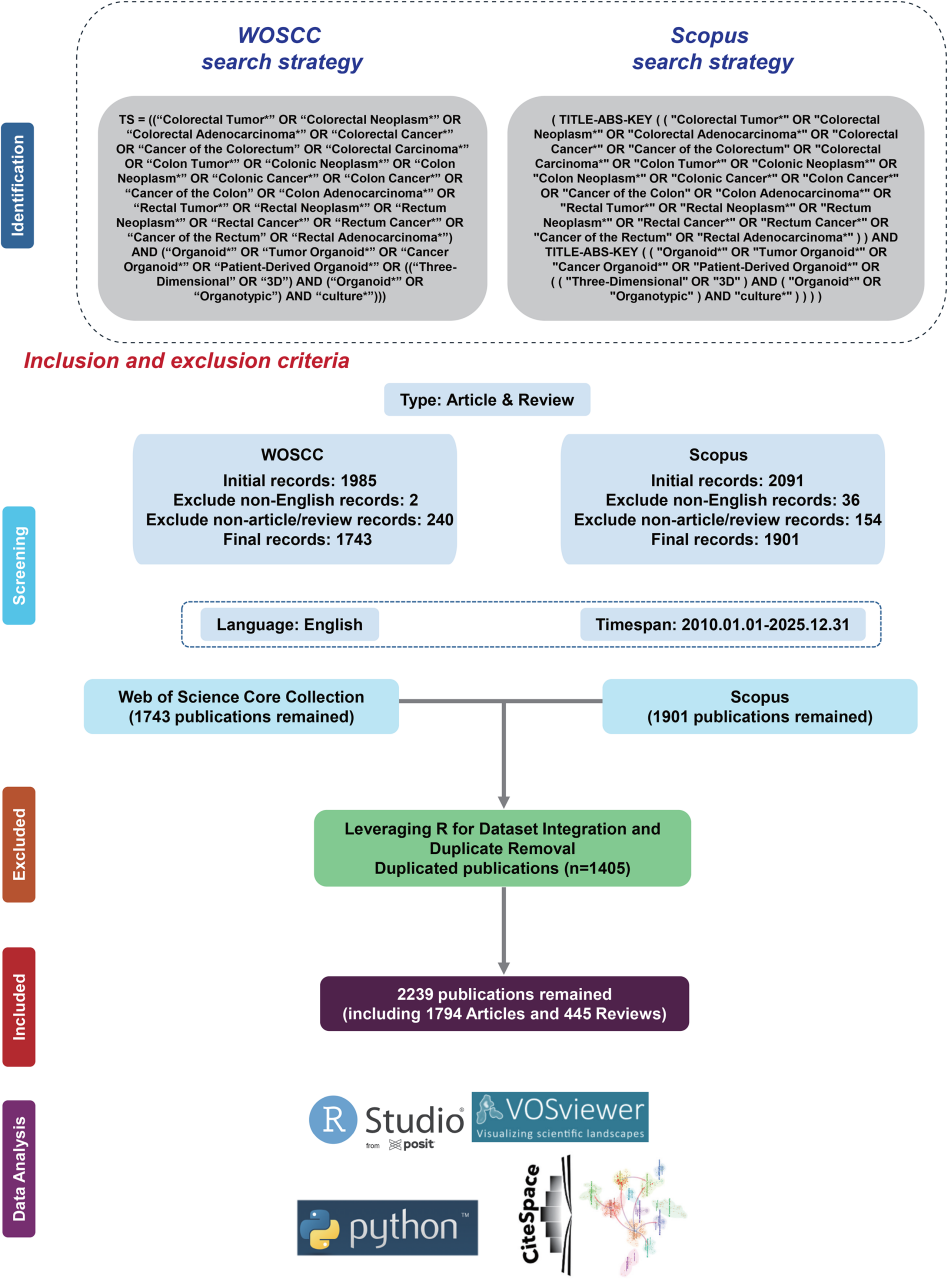
Flowchart of the study.

We developed a comprehensive search strategy focusing on organoids and colorectal cancer, as shown in **Figure 1**. Early 2026 publications were still under continuous updates and incomplete database coverage at the time of retrieval. To ensure dataset integrity and consistency, and avoid bias caused by incompletely indexed records, we only included literature fully indexed and incorporated by December 31, 2025. Literature types were limited to articles and reviews. A final total of 2,239 English publications dated from January 1, 2010, to December 31, 2025 were included. We performed data collection, screening, and data analysis in adherence to the PRISMA systematic review protocol (26). The detailed search strategy is laid out in **Supplementary Table 1**.

### 2.3 Bibliometric analysis and visualization

The Bibliometrix R package (v5.2.1) served as the primary tool for conducting descriptive and quantitative bibliometric analyses to characterize publication trends, productivity of authors, institutions and journals, collaboration networks, citation metrics of top-cited articles, and theme evolution trends (27). CiteSpace (v6.4.R1) was used for the dual map overlay of journals analysis, high-frequency keyword clustering, and burst detection of cited references, while VOSviewer (v1.6.20) was employed to visualize journal co-citation networks (28–30). Additionally, Python’s pandas and matplotlib packages were used for plotting.

## 3 Results

### 3.1 Descriptive bibliometric characteristics and publication trends

**Figure 2:**
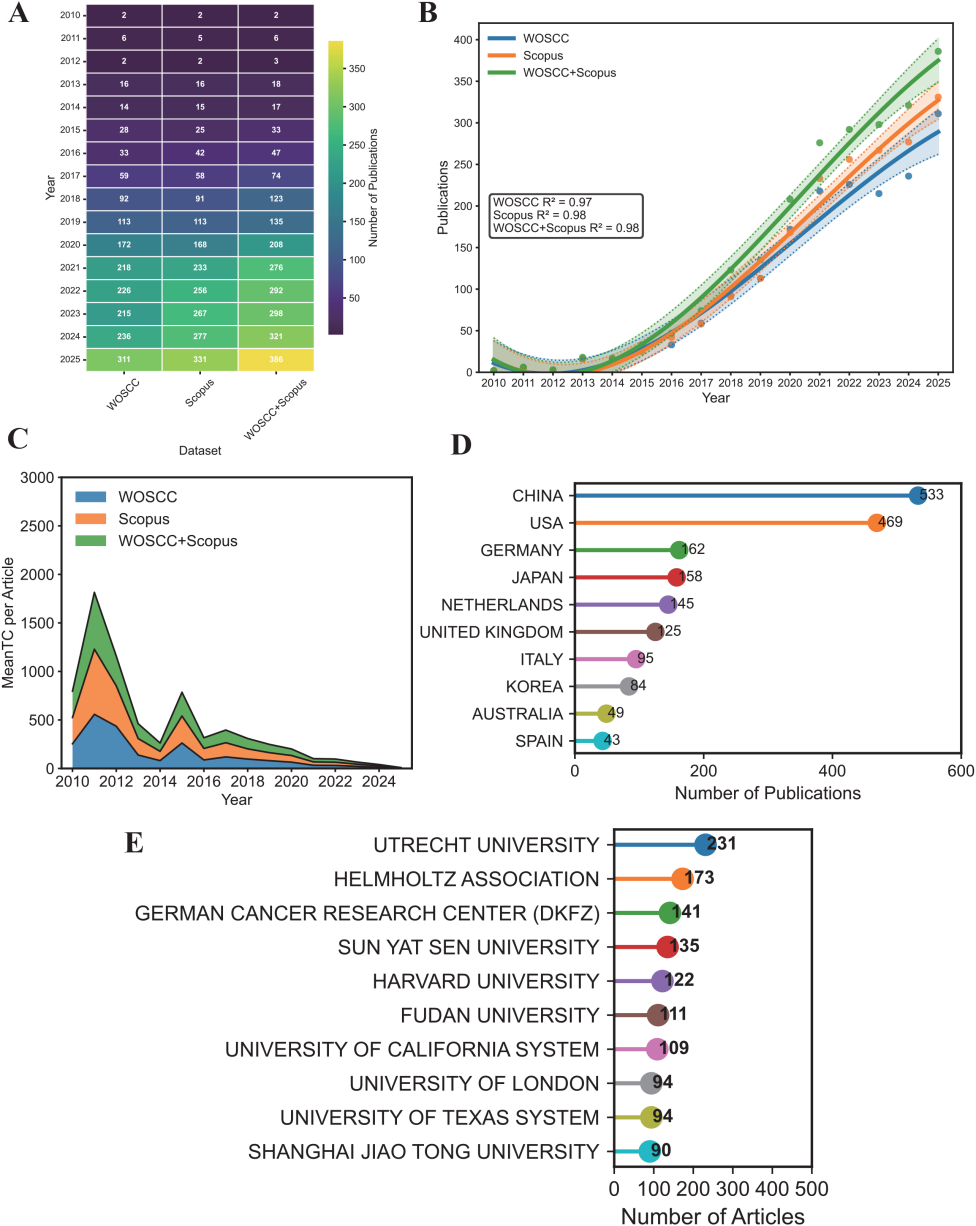
Publication trends and geographic distribution. **(A)** Number of publications in different databases. **(B)** Annual publication trends with fitted regression curves. **(C)** Annual trends in mean total citations per article. **(D)** Top 10 countries contributing to publications. **(E)** Top 10 institutions contributing to publications.

The final dataset was derived by merging the Web of Science Core Collection (WOSCC) and Scopus databases, with the merged data exhibiting trends consistent with those of the individual databases. After removing duplicates and excluding irrelevant articles in accordance with the predefined criteria, a total of 2,239 publications (including 1,794 articles and 445 reviews) were included. The corpus encompasses a substantial body of references, Keywords Plus, and author keywords from 2010 to 2025 (**Table 1**; **Supplementary Figure 1**). International collaboration accounted for 31.8% of publications, with an average of nearly eleven co-authors per document. An annual growth rate of 42.03% indicates that this field is in a phase of extremely rapid expansion. It is noteworthy that the proportion and average annual growth rate of internationally collaborative papers in the two databases are nearly identical. As shown in **Figure 2A**, only 2 papers were published in 2010, whereas this number reached 386 in 2025, representing more than a one-hundred-fold increase.

**Table 1:** Descriptive bibliometric statistics of datasets.

Description	WOSCC	Scopus	WOSCC+Scopus
Timespan	2010:2025	2010:2025	2010:2025
Sources (Journals, Books, etc)	471	507	601
Documents	1743	1901	2239
Annual Growth Rate %	40	40.58	42.03
Document Average Age	4.4	4.28	4.35
Average citations per doc	44.11	46	45.73
References	70623	12483	74527
Keywords Plus (ID)	3439	13415	9511
Author’s Keywords (DE)	3101	3506	3894
Authors	14753	15608	18498
Authors of single-authored docs	11	22	27
Single-authored docs	11	23	28
Co-Authors per Doc	11.5	11.7	11.2
International co-authorships %	32.01	32.61	31.8
article	1450	1599	1794
review	293	302	445

As illustrated in **Figure 2B**, curve fitting was performed on the annual publication counts. The fitted curve closely followed the observed annual publication counts, demonstrating a high goodness of fit (R^2^ = 0.98). The average number of citations per article for publications from 2010 to 2025 exhibits notable peaks in 2011 and 2015, suggesting that those years witnessed the publication of high-impact articles associated with potential technological breakthroughs (31, 32) (**Figure 2C**). Further details are available in **Supplementary Table 2**.

Analysis of national publication output showed that China ranked first with 533 publications, succeeded by the United States (469) and Germany (162) (**Table 2**; **Figure 2D**). The three leading contributors represented over 50% of global output. Multiple country publications (MCPs) reflected the number of collaborating authors from different nations and regions.

**Table 2:** Publication output and international collaboration patterns of top 10 countries.

Country	Articles	Articles %	SCP	MCP	MCP %
CHINA	533	23.8	435	98	18.4
USA	469	20.9	331	138	29.4
GERMANY	162	7.2	89	73	45.1
JAPAN	158	7.1	128	30	19
NETHERLANDS	145	6.5	84	61	42.1
UNITED KINGDOM	125	5.6	56	69	55.2
ITALY	95	4.2	54	41	43.2
KOREA	84	3.8	75	9	10.7
AUSTRALIA	49	2.2	25	24	49
SPAIN	43	1.9	26	17	39.5

Utrecht University in the Netherlands led institutional output with the highest number of publications (n=231). Other top contributors included the Helmholtz Association (173), the German Cancer Research Center (DKFZ) (141), Sun Yat-sen University (135), Harvard University (122), and Fudan University (111). Additional high-output institutions included the University of California System (109), the University of London (94), the University of Texas System (94), and Shanghai Jiao Tong University (90) (**Supplementary Table 3**; **Figure 2E**). In the realm of conventional scholarly institutions, Utrecht University stands out for its prominent research output. The results highlight a worldwide research landscape in the field of colorectal cancer organoids, with scholarly input originating from a diverse array of global institutions.

### 3.2 Visualized analysis of the dual-map overlay of journals

**Figure 3:**
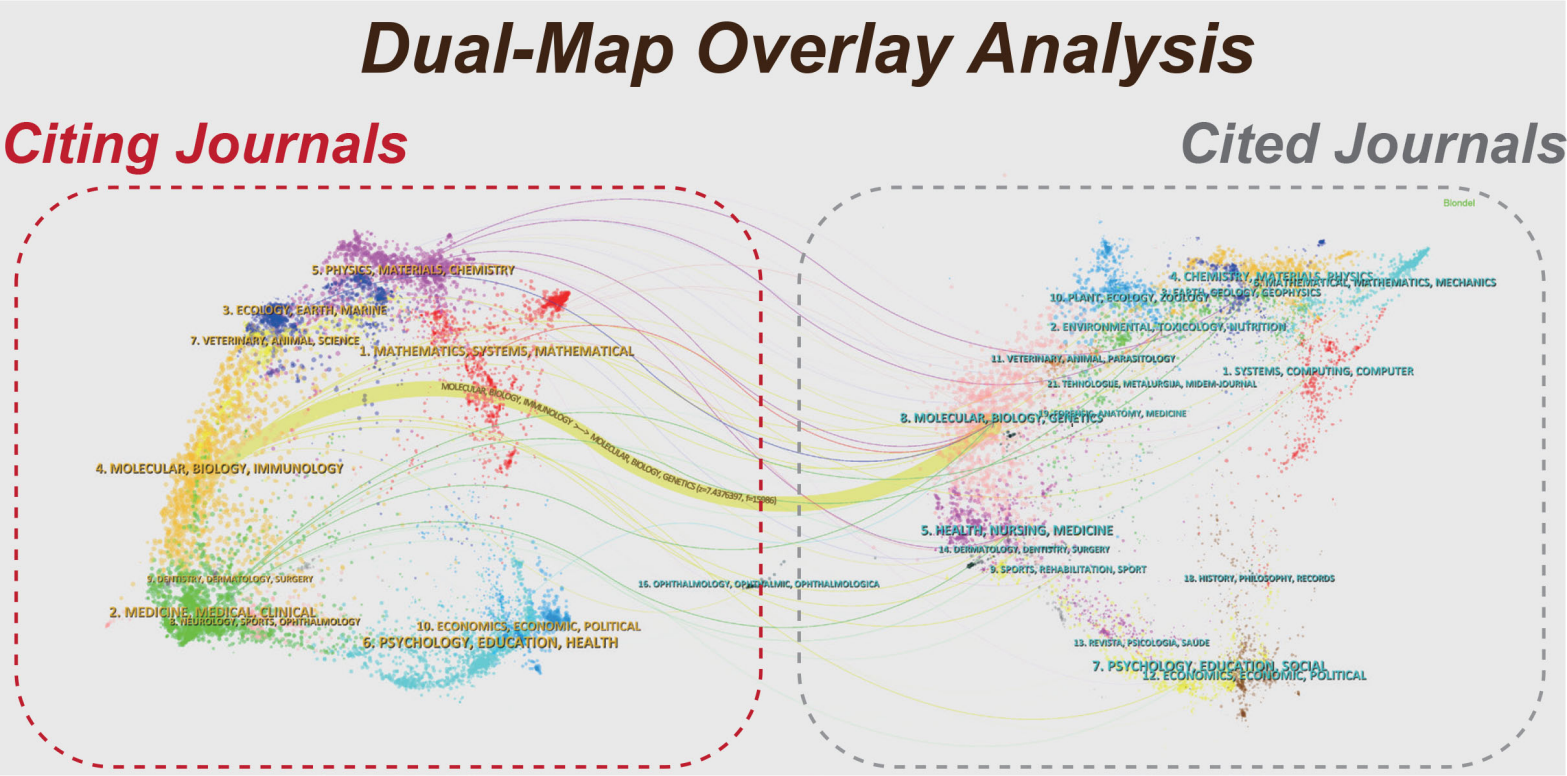
Dual-map overlay of journals in colorectal cancer organoid research.

The dual-map overlay of journals can reveal the research evolution across multiple disciplinary fields and capture the information flow at the journal level. As shown in **Figure 3**, the left portion corresponds to citing journals, and the right portion stands for cited journals. The colored curves in the Figure denote citation paths. The yellow path indicated that articles published in journals of Molecular/Biology/Genetics were frequently cited by journals in Molecular/Biology/Immunology. This analysis revealed the cross-citation relationships among multidisciplinary journals, reflecting the interdisciplinary collaboration and knowledge dissemination within the research field.

### 3.3 Analysis of author, country and journal productivity

**Figure 4:**
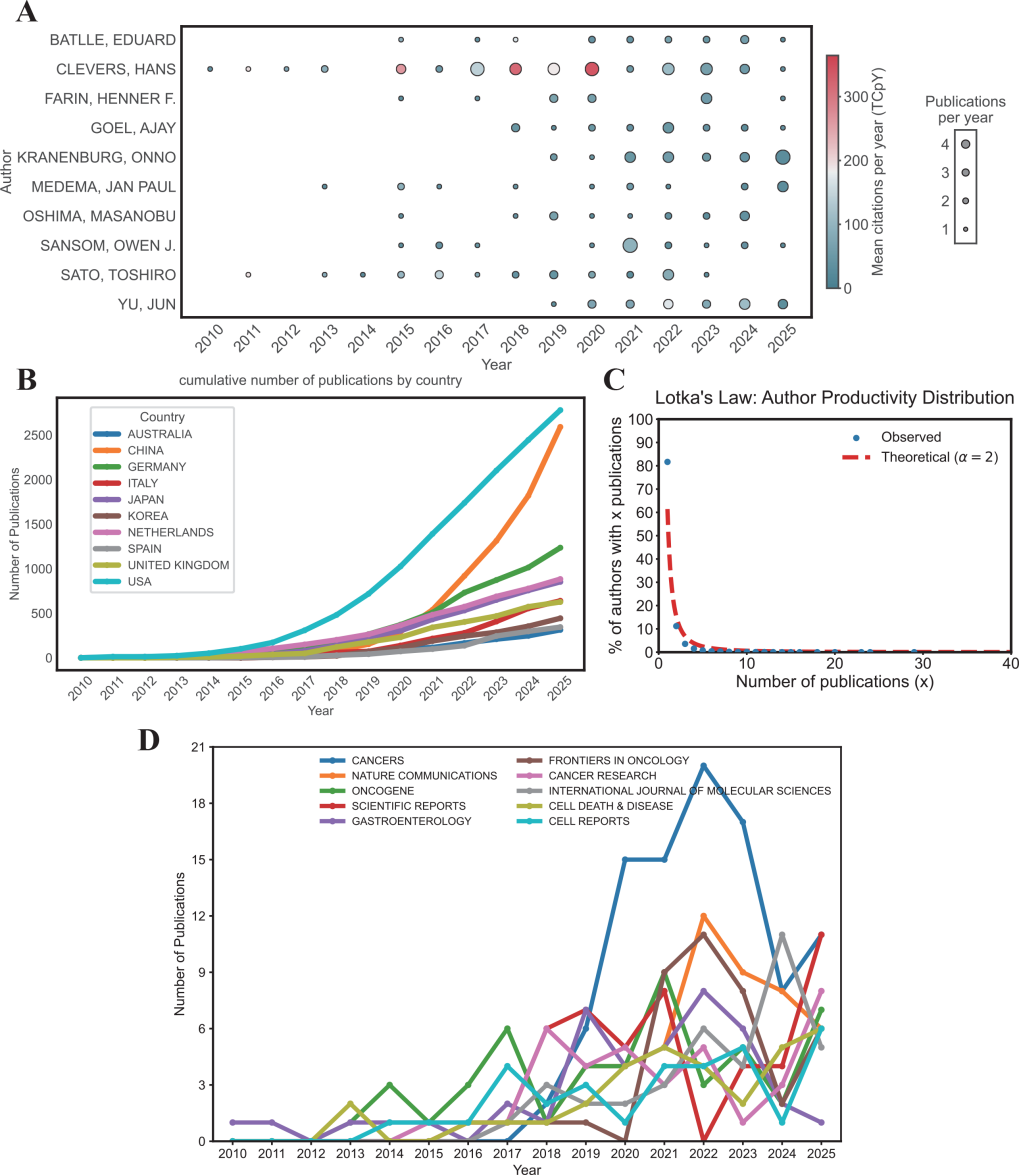
Analysis of author, country, and journal productivity. **(A)** Annual publication trends of the top 10 authors. **(B)** Cumulative publication trends of the top 10 countries. **(C)** Author productivity through Lotka's law. **(D)** Annual publication trends of the top 10 journals.

Author productivity and impact analyses revealed that Hans Clevers was the most productive author (NP = 58, TC = 18291) and held the highest h-index, g-index, and m-index (**Table 3**). Authors’ production over time showed that Hans Clevers has maintained consistent output and high mean citations per year since 2010, with particularly notable increases in 2018 and 2020 (**Figure 4A**). Additionally, Onno Kranenburg ranked second in publication output (NP = 29) and held the second-highest g-index, showing a significant surge in productivity in recent years. By utilizing Lotka’s Law, the analysis found that 81.7% of authors contributed only one publication (**Figure 4C**).

**Table 3:** Top 10 authors by quantitative academic metrics.

Author	h_index	g_index	m_index	TC	NP	PY_start
CLEVERS HANS	41	58	2.412	18291	58	2010
SATO TOSHIRO	18	24	1.125	8296	24	2011
SANSOM OWEN J.	17	20	1.417	1095	20	2015
YU JUN	17	23	2.125	2131	23	2019
GOEL AJAY	15	18	1.667	698	18	2018
KRANENBURG ONNO	13	29	1.625	887	29	2019
VOEST EMILE E.	13	14	1	3124	14	2014
VAN DE WETERING MARC	12	13	0.8	5282	13	2012
BATLLE EDUARD	11	15	0.917	3134	15	2015
FARIN HENNER F.	11	14	0.917	928	14	2015

Analysis of the cumulative number of publications by country (**Figure 4B**) revealed that the United States (USA) has consistently ranked first in terms of cumulative publications in this field from 2010 to 2025. Since 2010, the number of publications from all countries has shown a steady upward trend, with a marked acceleration in growth after 2015. Particularly notable increases were observed for China and the United States. Quantitative academic metrics at the journal level (**Table 4**) and annual publication trends (**Figure 4D**) collectively revealed the influence characteristics and output dynamics of core journals in this field. In terms of academic influence, Nature Communications ranked at the forefront with a comprehensive performance of h-index = 29, g-index = 51, and m-index = 2.636, boasting a total citation count of 3502 and 51 cumulative publications since its debut in 2016. The annual publication output of journals showed significant fluctuations over time. Cancers recorded the highest total number of publications among all journals, while *Nature* had the highest total citation count.

**Table 4:** Top 10 journals by quantitative academic metrics.

Source	h_index	g_index	m_index	TC	NP	PY_start
NATURE COMMUNICATIONS	29	51	2.636	3502	51	2016
GASTROENTEROLOGY	28	41	1.647	6452	41	2010
CANCERS	25	35	2.778	1664	94	2018
NATURE	23	25	1.533	9868	25	2012
ONCOGENE	23	40	1.643	1645	49	2013
CANCER RESEARCH	21	37	1.75	1426	38	2015
SCIENTIFIC REPORTS	20	34	1.818	1220	47	2016
CELL REPORTS	19	33	1.462	1874	33	2014
CELLULAR AND MOLECULAR GASTROENTEROLOGY AND HEPATOLOGY	19	24	2.111	791	24	2018
JOURNAL OF EXPERIMENTAL & CLINICAL CANCER RESEARCH	18	29	1.8	890	32	2017

### 3.4 Collaboration analysis

In order to assess the consistency and reliability of bibliographic data for visualizing academic partnerships, collaboration networks were developed at the institutional, national, and individual author levels utilizing both the Scopus and WOSCC databases. Collaboration networks of authors, countries, and institutions were visualized using the Bibliometrix R package (v5.2.1), confirming the stability and complementarity of the two databases in revealing collaborative network structures. While both databases consistently identified major collaborative groups, slight discrepancies in local collaborations suggest the value of combining data from multiple sources.

**Figure 5:**
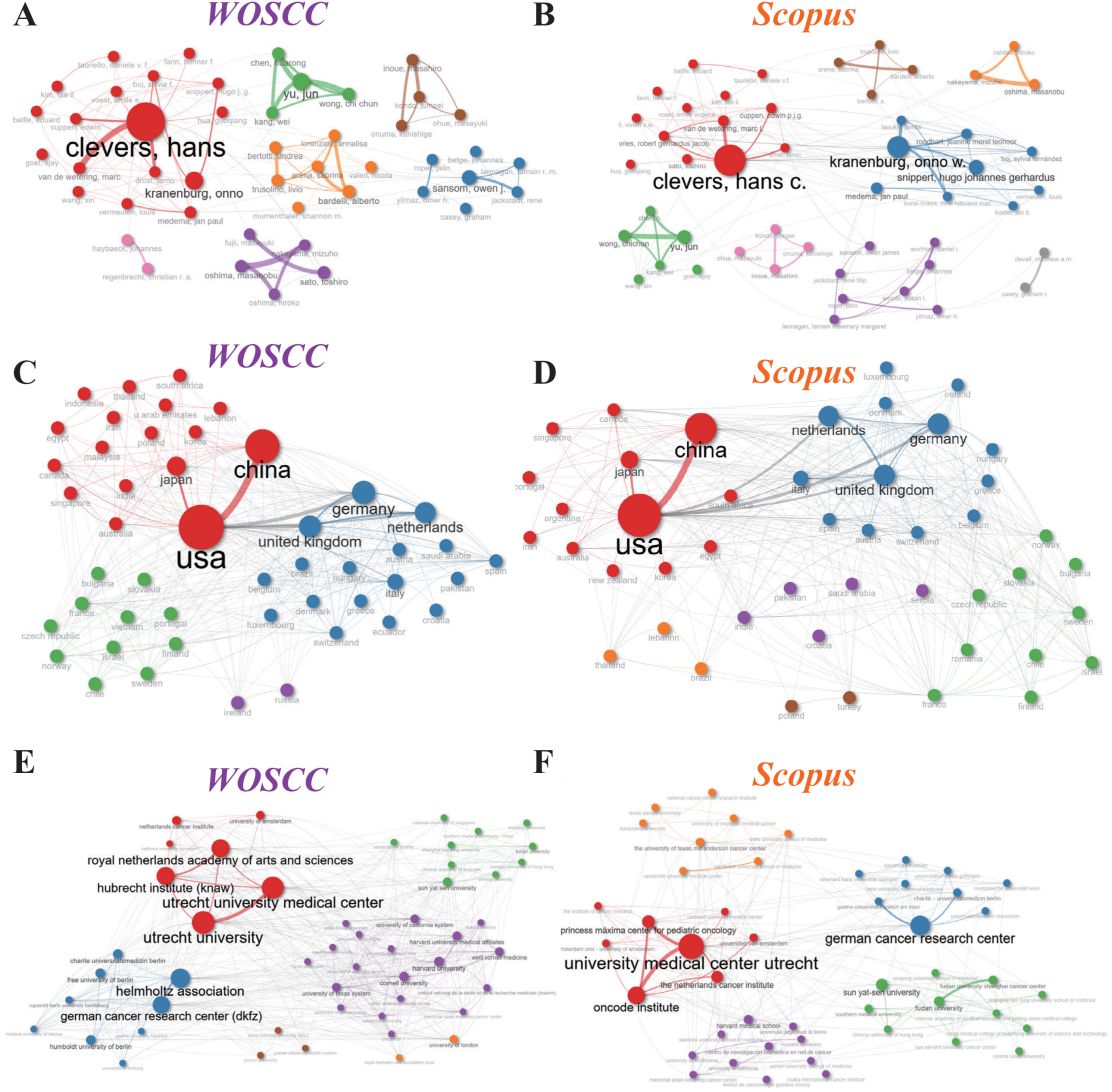
Collaboration networks at the author, country, and institutional levels. **(A)** Author collaboration network of WOSCC. **(B)** Author collaboration network of Scopus. **(C)** Country collaboration network of WOSCC. **(D)** Country collaboration network of Scopus. **(E)** Institutional collaboration network of WOSCC. **(F)** Institutional collaboration network of Scopus.

Author collaboration networks revealed distinct co-authorship clusters, with the size of the nodes determined by author centrality and the line thickness indicating the strength of the connection. The network topology indicated that in both the WOSCC network and the Scopus network (**Figures 5A, B**), Hans Clevers exhibited extremely high centrality, demonstrating his leadership role in thematic research development. Onno Kranenburg followed closely as a prominent node. However, the overall structure remained characterized by several smaller, closed sub-clusters with strong geographical localization. This structural feature reflects limited extensive collaboration across different author communities.

The national collaboration network revealed how countries collaborate in colorectal cancer organoid research (**Figures 5C, D**). Several nations stood out as major contributors. Analyses from both WOSCC and Scopus showed that the USA appeared as the largest central node, while China and Japan both maintained close collaboration with the USA. European nations like the UK, Germany, and the Netherlands exhibited strong internal collaboration, forming distinctive European industrial clusters and establishing cooperative relationships with the USA.

The institutional collaboration map revealed unique patterns in global research center connectivity (**Figures 5E, F**). In both the WOSCC and Scopus networks, institutions centered around Utrecht, Netherlands, formed the largest collaborative networks. WOSCC data showed KNAW (Hubrecht Institute/Royal Netherlands Academy of Arts and Sciences), Utrecht University, and UMC Utrecht forming the densest core. This centrality was further validated in Scopus data, with Oncode being another central node. Additionally, the German Cancer Research Center (DKFZ) was another key node in Europe.

### 3.5 Citation analysis

**Figure 6:**
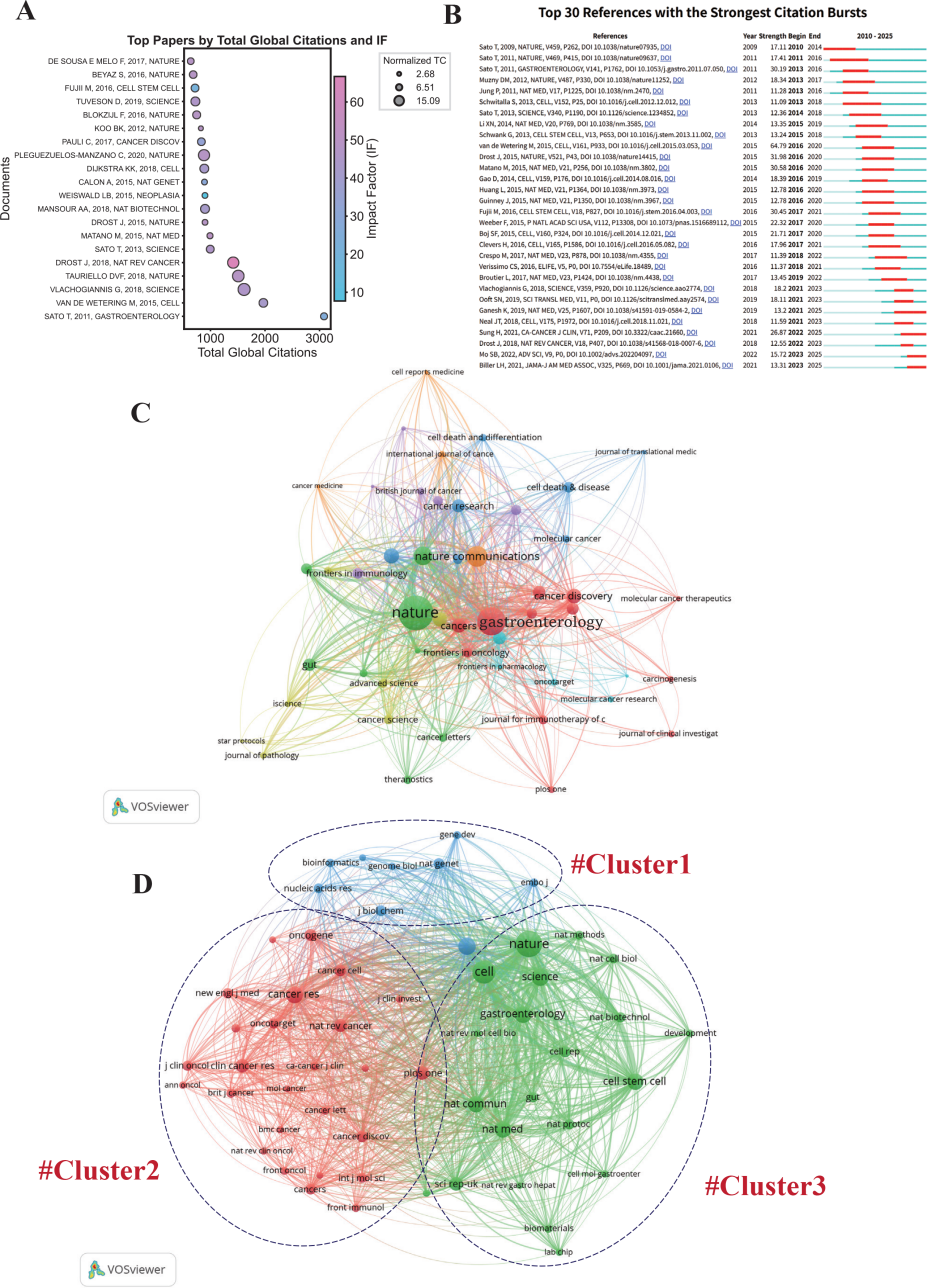
Citation and co-citation analysis. **(A)** Top papers by total global citations and impact factor. **(B)** Top 30 references with the strongest citation bursts. **(C)** Journal citation network. **(D)** Clustered journal co-citation network.

The top high-impact publications on colorectal cancer organoids, ranked by total citations and journal impact factor, are shown in **Figure 6A**. These highly cited papers exhibit total citations ranging from 600 to over 3,000, with journal IFs primarily concentrated between 20 and 60. The top 20 most-cited papers in colorectal cancer organoid research, covering basic publication information, DOI, global total citations, normalized citation counts, and journal impact factors, were summarized in **Table 5**. Despite not having the highest total citations, some papers in high-ranking journals exhibit excellent normalized citation counts and IF, underscoring their significant academic impact in the field.

**Table 5:** Top 20 cited papers.

Paper	DOI	Global total citations	Normalized TC	IF
SATO T, 2011, GASTROENTEROLOGY	10.1053/j.gastro.2011.07.050	3083	5.26	25
VAN DE WETERING M, 2015, CELL	10.1016/j.cell.2015.03.053	1970	8.01	42.5
VLACHOGIANNIS G, 2018, SCI.	10.1126/science.aao2774	1616	15.09	45.7
TAURIELLO DVF, 2018, NATURE	10.1038/nature25492	1510	14.10	48.4
DROST J, 2018, NAT REV CANCER	10.1038/s41568-018-0007-6	1417	13.23	66.7
SATO T, 2013, SCIENCE	10.1126/science.1234852	996	6.51	45.7
MATANO M, 2015, NAT MED	10.1038/nm.3802	991	4.03	49.9
DROST J, 2015, NATURE	10.1038/nature14415	900	3.66	48.4
MANSOUR AA, 2018, NAT BIOTECHNOL	10.1038/nbt.4127	900	8.40	41.6
WEISWALD LB, 2015, NEOPLASIA	10.1016/j.neo.2014.12.004	899	3.66	7.7
CALON A, 2015, NAT GENET	10.1038/ng.3225	890	3.62	28.9
DIJKSTRA KK, 2018, CELL	10.1016/j.cell.2018.07.009	887	8.28	42.5
PLEGUEZUELOS-MANZANO C, 2020, NATURE	10.1038/s41586-020-2080-8	881	13.00	48.4
PAULI C, 2017, CANCER DISCOV	10.1158/2159-8290.CD-16-1154	833	6.38	33.2
KOO BK, 2012, NATURE	10.1038/nature11308	824	2.68	48.4
BLOKZIJL F, 2016, NATURE	10.1038/nature19768	747	6.78	48.4
TUVESON D, 2019, SCI.	10.1126/science.aaw6985	723	8.48	45.7
FUJII M, 2016, CELL STEM CELL	10.1016/j.stem.2016.04.003	717	6.51	20.4
BEYAZ S, 2016, NATURE	10.1038/nature17173	683	6.20	48.4
DE SOUSA E MELO F, 2017, NATURE	10.1038/nature21713	638	4.89	48.4

Citation bursts reflected a significant change in the citation frequency of a cited document within a specific period (**Figure 6B**). Two early seminal papers by Toshiro Sato, Hans Clevers et al. published in Nature exhibited strong citation bursts, laying a critical foundational framework for subsequent research in this field. Notably, a paper by Marc van de Wetering, Hans Clevers et al. published in Cell showed the strongest citation burst of 64.79, suggesting its lasting and significant impact at the time of publication.

The top 20 co-cited journals ranked by total co-citations and total link strengths were presented in **Table 6**.

**Table 6:** Top 20 co-cited journals.

Source	Citations	Total link strength
nature	6179	311682
cell	4743	251779
science	2779	152118
p natl acad sci usa	2759	142918
cancer res	2560	132427
gastroenterology	2527	121257
nat commun	2445	141765
nat med	2359	139353
cell stem cell	2354	152355
sci rep-uk	1590	88617
clin cancer res	1580	78933
plos one	1567	76979
nat rev cancer	1460	72230
oncogene	1268	60022
cell rep	1179	71893
cancer cell	1166	59184
cancer discov	1130	67326
cancers	1046	55470
nat genet	1020	52175
j biol chem	1019	39746

Journal co-citation analysis is an important method for identifying influential journals within a specific research field. Journals with at least 10 citations and journals with 400 or more co-citations were presented in **Figures 6C**, **D**. Connections between nodes represent the strength of collaboration or citation with thicker lines indicating stronger associations. Co-cited journals are partitioned into three clusters distinguished by different colors. Cluster 1 (blue cluster) focused on genomics and molecular biology; Cluster 2 (red cluster) was dominated by oncology journals addressing cancer mechanisms and therapy; Cluster 3 (green cluster) was anchored in leading general journals integrating stem cell research, translational medicine, and basic life sciences.

### 3.6 Keywords analysis

**Figure 7:**
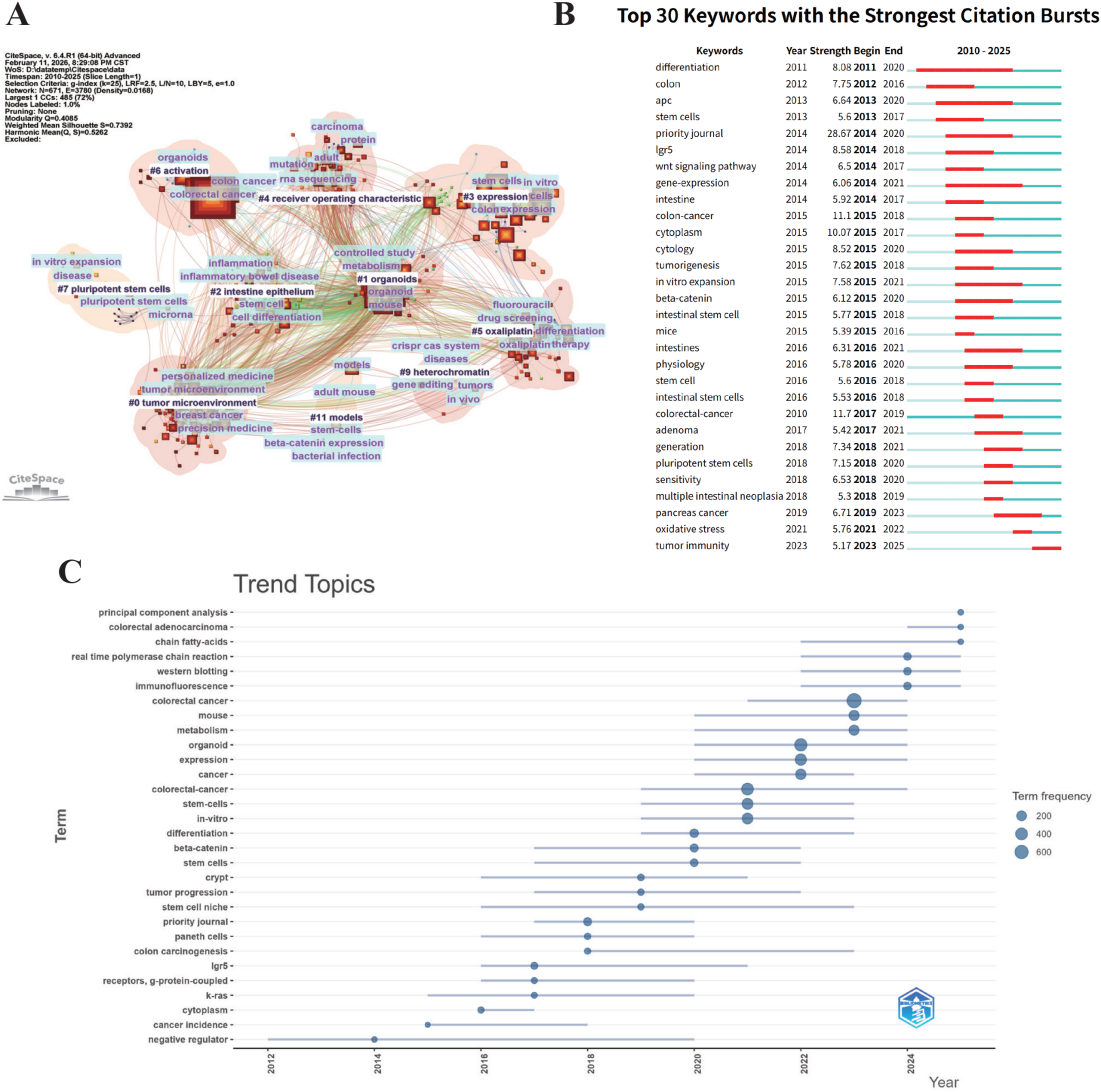
Keyword analysis. **(A)** Keyword clustering map. **(B)** Top 30 keywords with the strongest citation bursts. **(C)** Temporal trends in keyword evolution.

Keywords provided by authors represent the core themes and content of articles. Based on the log-likelihood ratio (LLR) algorithm, cluster analysis of the imported keywords was conducted utilizing CiteSpace, generating a total of 10 clusters (**Figure 7A**). The modularity Q value > 0.3 (Q = 0.4085) suggests a significant clustering structure, the weighted mean silhouette S value > 0.5 indicates reasonable clustering results, and S > 0.7 (S = 0.7392) represents highly significant clustering. Further cluster analysis of keywords identified three major thematic areas: core models and technical systems (e.g., #6 activation, #7 pluripotent stem cells, #11 models), tissue biology and disease pathogenesis (e.g., #2 intestine epithelium, #3 expression cells), and clinical application and translational research (e.g., #4 receiver operating characteristic, #5 oxaliplatin). The top 30 keywords and their corresponding frequencies are shown in **Supplementary Table 4**.

Keyword burst analysis identifies keywords with a surge in frequency or intensity over a short period, revealing the evolution and shifts of research hotspots (**Figure 7B**). By combining this analysis with the temporal trend graph of keywords (**Figure 7C**), the evolutionary trajectory of colorectal cancer organoid research was illustrated. In the early phase (2010-2018), keywords such as differentiation, apc, wnt signaling pathway, and β-catenin indicated a primary focus on basic mechanisms and the establishment of traditional organoid models. This foundational research orientation was further corroborated by the high-frequency terms shown in **Figure 7C**, including k-ras and g-protein-coupled receptors. After 2018, the research focus gradually shifted toward clinical translation and the tumor microenvironment. The emergence of burst keywords such as sensitivity and tumor immunity in **Figure 7B**, together with the high frequency of terms such as stem cell niche and metabolism in **Figure 7C**, suggested that research during this phase has expanded from mechanistic exploration into contexts more relevant to clinical application.

### 3.7 Analysis of thematic evolution

**Figure 8:**
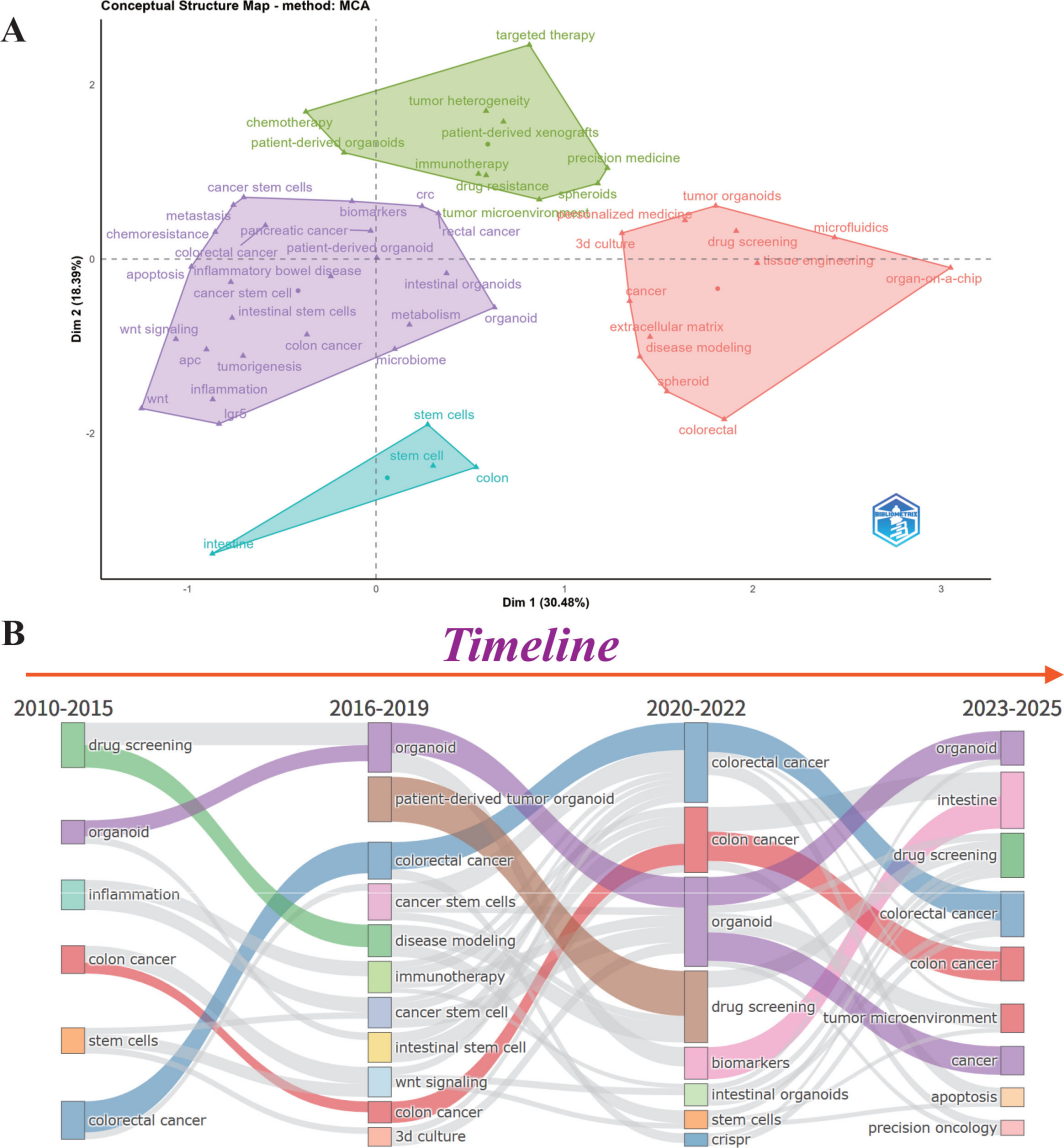
Analysis of thematic evolution. **(A)** Conceptual structure map via multiple correspondence analysis (MCA). **(B)** Timeline of thematic evolution.

Multiple correspondence analysis (MCA) was employed to visualize the conceptual framework of the literature by clustering interrelated terms. It primarily identified three distinct dimensions (**Figure 8A**). The green area at the top contained terms related to tumor treatment. The purple area on the left encompassed terms associated with molecular mechanisms, pathogenesis and pathological processes. The red area on the right covered *in vitro* model construction.

As shown in **Figure 8B**, the Sankey diagram illustrates the evolution of research themes from 2010 to 2025, with each node representing a theme and flow width indicating publication volume. Organoid, colorectal cancer, drug screening, and colon cancer served as core themes throughout the timeline. Disease modeling emerging in 2016–2019 was closely linked to early drug screening (2010–2015), and patient-derived tumor organoids (PDTOs) arising in 2016–2019 showed a strong correlation with drug screening in 2020–2022, indicating the progressive development of colorectal cancer organoid research toward clinical application. Stem cell research has gradually branched into more specific directions since 2016–2019, such as cancer stem cells and intestinal stem cells. Both immunotherapy introduced in 2016–2019 and crispr emerged in 2020–2022 have been integrated into colorectal cancer research, expanding novel therapeutic strategies for the disease. The emergence of precision oncology and tumor microenvironment as new research focuses during 2023–2025 revealed the promising application prospects of colorectal cancer organoids in precision medicine.

### 3.8 Validation analysis of non-review articles

**Figure 9:**
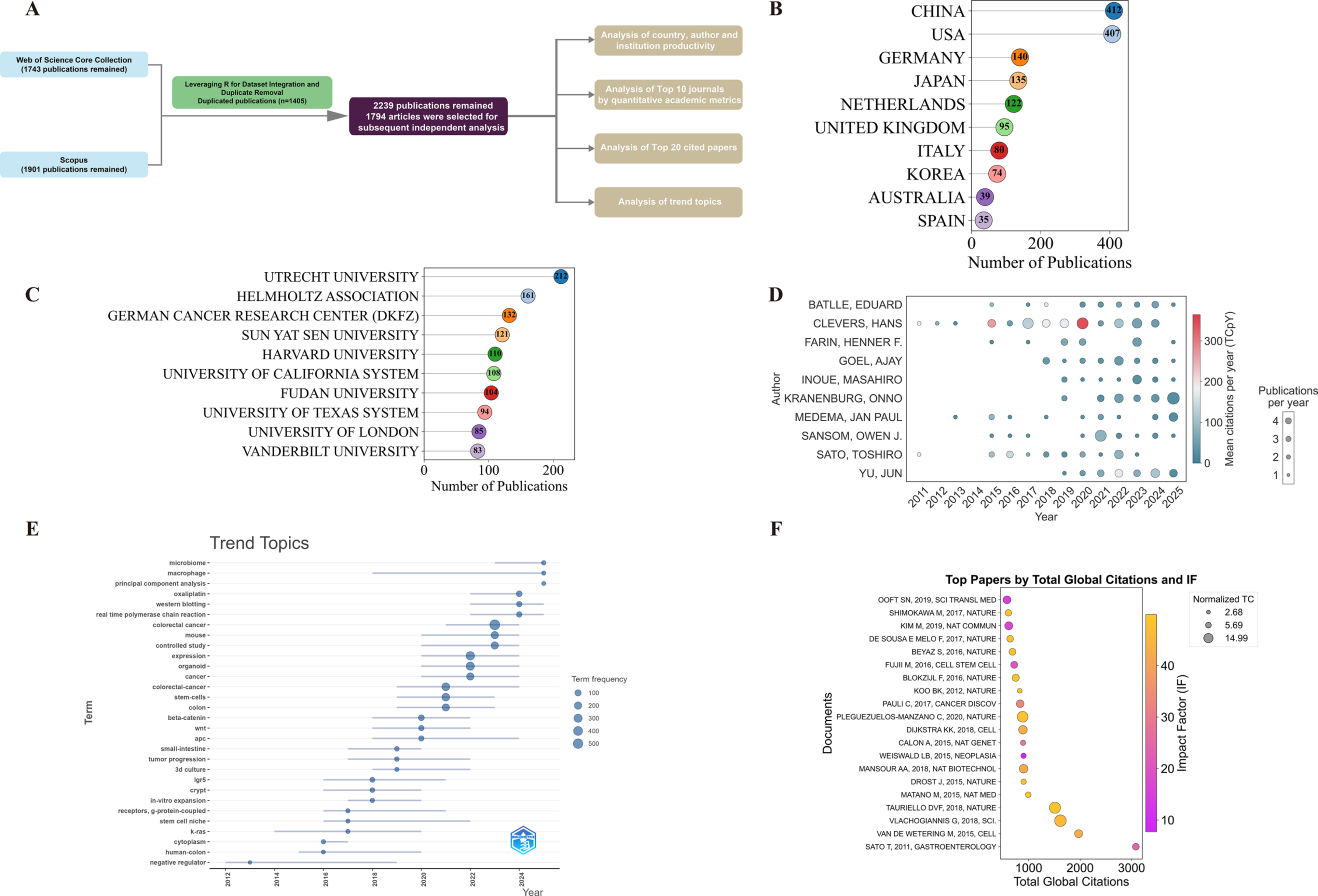
Validation analysis of non-review articles. **(A)** Flowchart of the validation analysis. **(B)** Top 10 countries contributing to publications. **(C)** Top 10 institutions contributing to publications. **(D)** Annual publication trends of the top 10 authors. **(E)** Temporal trends in keyword evolution. **(F)** Top papers by total global citations and impact factor.

To prevent review articles from interfering with the core research signals derived from primary studies, we conducted a separate validation analysis exclusively on non-review articles (**Figure 9**; **Supplementary Tables 5–9**). After excluding the review articles, although the publication outputs of countries, institutions, and authors decreased, the overall trends remained largely unchanged. Specifically, the country rankings were entirely consistent with the pre-exclusion results.

Notably, in the analysis of institutional output, the 10th position, previously held by Shanghai Jiao Tong University, was replaced by Vanderbilt University, while the University of California System and the University of Texas System both advanced by one rank; Trend Topic Analysis demonstrates that the microbiome and macrophage are also emerging hot research topics, involving immunology and microbiology; Among the top 20 publications, only one review article authored by Jarno Drost and Hans Clevers is included in the five papers with significantly higher normalized total citations than the remaining 15 publications.

## 4 Discussion

### 4.1 Bibliometric trend summary

Research on colorectal cancer organoids has grown explosively over the past sixteen years. The period leading up to 2014 was marked by a modest growth in publications, during which no distinctive patterns or surges were observed. However, from 2015 to 2025, the field witnessed a sharp increase in publications. This indicates that the field of colorectal cancer organoids will grow more active and robust in the future. China and the United States have emerged as leading contributors among the top 10 productive countries with 44.7% of total publications. High incidence and mortality of colorectal cancer in China and the US have strongly promoted relevant studies (33, 34). Furthermore, such progress was driven by robust research capacity and sustained funding support, while the marked growth in recent years may also be facilitated by regulatory advancements such as the FDA Modernization Act 2.0.

The top three institutions by publication volume are from Germany and the Netherlands. Notably, the most influential author is from the Netherlands, indicating that individual leadership plays a vital role in developing the field. Although the United States and China had the largest number of MCPs, their MCP ratios (MCP/articles) were merely 29.4% and 18.4% respectively, far lower than those of the United Kingdom (55.2%) and Germany (45.1%). This finding suggests that most research was conducted domestically, and international collaboration needs to be strengthened.

Gastroenterology (IF = 25.9, Q1) and Nature (IF = 48.5, Q1) are frequently cited journals. This suggests their work carries considerable academic influence and recognition, and researchers should closely monitor these journals. Citation-burst references can reveal research hotspots. In this study, such references covered the following aspects: drug screening, tumor microenvironment, precision medicine, organoid model construction, and tumor immunity. Four remained bursty through 2025 with two detailed below. One study by Karuna Ganesh et al. demonstrated that an organoid-based platform could investigate the drug sensitivity of clinically isolated rectal cancer strains, providing a reliable tool for efficient drug screening (35). In addition, Shaobo Mo et al. reported that patient-derived organoids from colorectal cancer liver metastasis could predict chemotherapy responses, directly supporting the precision oncology and personalized treatment strategies (36).

Keywords are crucial for identifying core research areas. Integrating them with thematic evolution can uncover the developmental trajectory and emerging trends of the field. This study revealed that research hotspots were mainly concentrated in disease modeling, drug screening, tumor microenvironment, and personalized medicine.

### 4.2 Exploration of research trends and hotspots

#### 4.2.1 Disease modeling approaches and applications

**Figure 10:**
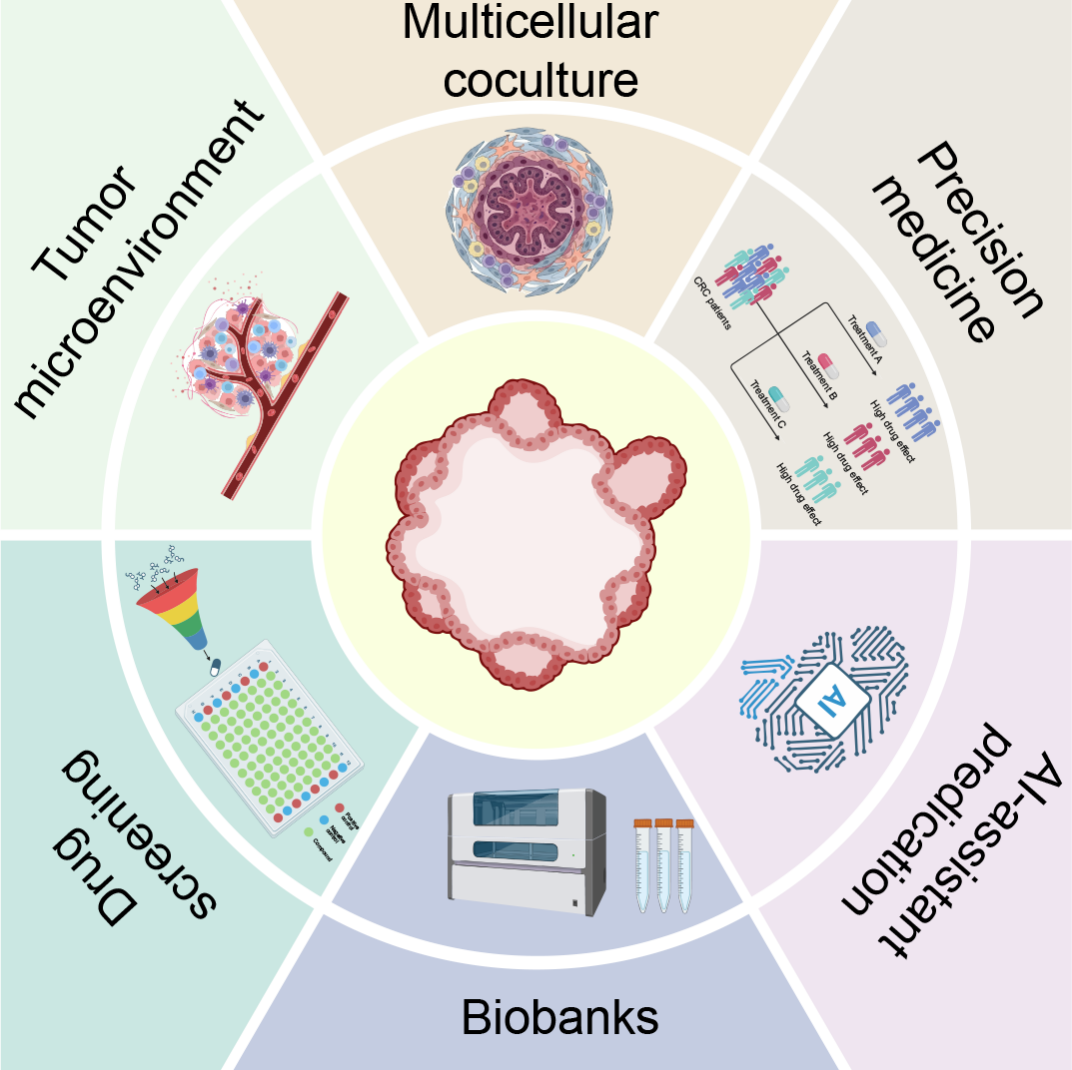
Current hotspots in colorectal cancer organoid research. Created in BioRender. ZHANG, X. Y. (2026) https://BioRender.com/2maut4r.

The current research hotspots in colorectal cancer organoids are shown in **Figure 10**. Traditional tumor research models include cell lines and animal models. However, due to the lack of *in vivo* cellular characteristics or interspecies differences, they often exhibit limitations in the study of drug resistance mechanisms and clinical guidance. In 2009, the team led by Hans Clevers first successfully generated miniature intestinal organoids from single Lgr5+ adult stem cells in mice (37). In 2011, this team established colorectal cancer organoid models using tumor tissue from colorectal cancer patients, marking the first expansion of organoid technology to the field of oncology (31). However, this model had limitations in simulating the intact immune system, tumor microenvironment (TME), and organ–organ interactions (38). Numerous studies have attempted to employ coculture methodologies to bring PDTOs closer to authentic *in vivo* conditions. PDTOs can accurately recapitulate the 3D tumor architecture, heterogeneity, and drug response of tumors, indicating their translational potential from bench to bedside in predicting clinical therapeutic responses (9).

Nevertheless, inherent limitations are inevitable for any model whose composition and function fail to recapitulate authentic *in vivo* conditions (39–43). Researchers have attempted to construct tumor models integrating stromal and immune components to investigate and predict patients’ therapeutic responses to T cell checkpoint blockade or immune cell therapies (44–46). However, these models failed to recapitulate the native tumor architecture and fully preserve the diversity of immune and non-immune stromal components. Recent advances in 3D bioprinting have made it possible to establish organoid models with well-defined structure, spatial control, and faithful integration of tumor and stromal components (47, 48).Additionally, endothelial cells, fibroblasts, and immune cells can be incorporated in organ-on-a-chip systems to recapitulate a comparatively complete tumor microenvironment (49, 50). *In vitro* vascularized microtumors (VMTs) serve as a comparatively typical model in colorectal cancer research (51). Assessments of drug delivery and penetration have also been realized within comparable colorectal cancer organ-on-a-chip platforms (52, 53). Furthermore, conventional PDTOs often fail to preserve cellular states associated with regeneration and fetal-like transcriptional programs. Xiong L et al. established a chemically defined patient-derived organoid system that enables long-term expansion of CRC cells, while recapitulating and maintaining the dynamic plasticity of tumor cells (54). Taken together, 3D bioprinting, organ-on-a-chip technology, and optimized chemically defined systems have significantly enhanced the structural and functional fidelity of CRC organoids, bolstering their reliability as translational platforms for personalized medicine.

#### 4.2.2 Drug screening and therapeutic evaluation

From the perspective of our analysis, colorectal cancer organoids have been widely applied in drug screening and have emerged as a prominent research hotspot. Several studies have evaluated the clinical predictive performance of patient-derived organoids in colorectal cancer. A prospective cohort study by Roodhart and colleagues included 232 patients with metastatic colorectal cancer. Patient derived organoids showed strong predictive accuracy for the fluorouracil/oxaliplatin regimen, with a positive predictive value of 0.78, a negative predictive value of 0.80, and an AUROC between 0.78 and 0.88. These measures correlated significantly with progression free survival and overall survival (P = 0.016 and 0.049, respectively) (55). Another prospective clinical study by Ooft et al. found that organoid predictive performance depended strongly on the drug class. Their results indicated that the drug dependent nature of these models is a key consideration (56).

Drug resistance mechanisms of colorectal cancer have been increasingly investigated using patient-derived organoid models. Huismans and coauthors compared 35 patient derived organoid lines from previously treated versus treatment naïve individuals with metastatic colorectal cancer. Organoids from pretreated patients carried a higher mutational burden and more structural variants (57). In addition, the oxaliplatin resistant phenotype remained stable in these organoids, highlighting how deeply treatment history influences tumor biology. Separately, Liu et al. confirmed that the circular RNA hsa_circ_0020095 regulates chemoresistance in colorectal cancer. Knockdown of this RNA reversed resistance to 5-fluorouracil and oxaliplatin in patient derived organoids, offering a potential target to overcome chemotherapy resistance (58). In summary, colorectal cancer organoids can effectively predict the clinical efficacy of drugs and elucidate tumor drug resistance mechanisms, providing a cutting-edge platform for the successful translation into personalized medicine and clinical drug development.

#### 4.2.3 Tumor microenvironment characterization

The tumor microenvironment in colorectal cancer is a three-dimensional, dynamic, and heterogeneous space comprised of multicellular and non-cellular stromal components, soluble regulatory factors, and physicochemical features. It functions as a core system orchestrating colorectal cancer cell proliferation, epithelial-mesenchymal transition (EMT), invasion and metastasis, immune evasion, as well as drug response and resistance (9, 59). Additionally, the tumor microenvironment is crucial for evaluating therapies reliant on TME interactions, particularly immunotherapy (60). Although tumor organoids do not inherently contain immune cells, this limitation can be overcome by introducing the air-liquid interface (ALI) technique or microfluidic organ-on-a-chip models (59, 61). For example, by incorporating these TME components, it has been demonstrated that cancer-associated fibroblasts (CAFs) drive MMP7-mediated invasiveness via an IL-1B autocrine loop and interact with tumor-infiltrating lymphocytes (TILs) to promote immune evasion through the upregulation of CD274 in CRC cells (59). Furthermore, tumor-reactive T cells can be enriched and selected by means of drug sensitivity and resistance screening assays in T cell-based therapies (62).

#### 4.2.4 Personalized medicine and clinical translation

PDTOs are also valuable tools for predicting individual responses to cancer treatment, as they retain patients’ genetic and epigenetic aspects (63). Weeber et al. showed that organoids derived from patients with metastatic colorectal cancer retained 90% of somatic mutations and exhibited a DNA copy number correlation coefficient of 0.89 relative to the original tumors, confirming their genetic fidelity (64). These organoids accurately predicted treatment responses in colorectal and gastroesophageal cancers, achieving a positive predictive value of 88% and a negative predictive value of 100% (65). Furthermore, they effectively forecasted radiotherapy sensitivity, supporting their utility in personalized treatment (66). Fujii et al. established a library of 55 colorectal tumor organoid lines and performed genetic characterization and niche factor-based optimization, achieving comprehensive genotype-phenotype analyses (67).

Colorectal cancer organoids, including rectal cancer, also predicted chemotherapy and radiotherapy responses. Biomarkers can also be used to measure the response of PDTOs to treatment. A study showed that gene mutations such as KRAS are linked to higher chemoradiotherapy resistance (35). One study analyzed the correlation with radiotherapy response in 19 colorectal cancer patients and developed a predictive model with 82% accuracy for sensitive patients and 92% accuracy for resistant patients (68).

Single-cell sequencing combined with patient-derived organoids (PDOs) can reveal the molecular heterogeneity of original tumors (69).Using single-cell data, Clinicians can leverage single-cell data to refine and optimize personalized treatment plans. By gaining detailed insights into the responses of various cell types within a patient’s tumor, they can precisely tailor treatment regimens to the tumor’s unique characteristics, thereby enhancing treatment efficacy and minimizing adverse side effects (70).

Moreover, the capacity of organoid models to recapitulate tumor heterogeneity and immune escape facilitated the evaluation of vaccines tailored to the unique tumor antigens of individual patients (71). Such platform contributed to the development of personalized vaccines and the enhancement of vaccine efficacy in clinical settings.

### 4.3 Progress in clinical trials

In recent years, the rapid advancement of organoid technology has led to remarkable progress in clinical trials within the realm of CRC treatment. Multiple prospective studies have confirmed that patient-derived organoids not only accurately recapitulate patient-specific drug responses *in vitro* but also hold promise as functional platforms for guiding clinical decision-making. The pioneering French multicenter Phase I/II trial, ORGANOTREAT-01, provided the first demonstration of the feasibility of integrating PDO-based drug screening into the clinical workflow within a large-scale prospective cohort (72). The accuracy of PDOs in predicting therapeutic efficacy has recently undergone rigorous validation in larger cohorts. A prospective Dutch study involving 232 patients with metastatic CRC demonstrated that, through optimized culture conditions, an overall PDO establishment rate of 52% could be achieved. The chemosensitivity assays for oxaliplatin and 5-fluorouracil exhibited high predictive accuracy, with a positive predictive value of 0.78 and a negative predictive value of 0.80, strongly supporting the potential of PDOs as a functional companion diagnostic tool (55).

In specialized therapeutic contexts such as hyperthermic intraperitoneal chemotherapy (HIPEC), organoids are also being employed to optimize regimen selection. Focusing on the poor-prognosis population with colorectal cancer peritoneal metastases, an Italian study utilized PDOs to simulate the hyperthermic perfusion environment for chemosensitivity testing. The findings indicated that mitomycin C, either alone or in combination with cisplatin, was superior to oxaliplatin at clinically relevant concentrations (73). Another study, conducted on 42 CRC organoids, further confirmed that HIPEC regimens containing mitomycin C achieved a mean tumor cell inhibition rate of 85.2% (95% confidence interval [CI] 80.4-89.9%), which was significantly higher than the 37.9% (95% CI 31.5-44.3%) observed with oxaliplatin-based regimens (74). Furthermore, in rare aggressive subtypes such as signet-ring cell carcinoma, organoid models have unveiled novel therapeutic targets. Research revealed that these tumors exhibit heightened sensitivity to regimens combining FOLFIRI with paclitaxel or vincristine, thereby charting a course for subsequent clinical trials (75). In summary, research on colorectal cancer organoids is transitioning from the early phase of model establishment to a new stage focused on validating clinical utility through large-scale, prospective clinical trials.

### 4.4 Challenges and future directions

Currently, CRC organoids still face several challenges including incomplete recapitulation of the tumor microenvironment, the lack of standardized protocols and reproducibility, stability issues throughout prolonged cultivation, and elevated expenses for massive implementation. In the future, CRC organoids should be integrated with multicellular co-culture systems that incorporate not only various immune cell types but also stromal and vascular components. 3D bioprinting technology and microfluidic technology will enhance model accuracy to better recapitulate the complex tissue microenvironment, and organ-on-a-chip systems hold great potential for large-scale personalized PDTO modeling and drug screening (76–78). Continuous efforts to optimize culture protocols, data sharing and validation methodologies will help address the standardization and reproducibility of organoid models. AI-driven monitoring systems can forecast nutritional demands and enable real-time modulation of culture conditions (79). Integration of such systems with automated platforms can not only improve stability but also reduce costs, thus rendering large-scale long-term culture more feasible.

### 4.5 Limitations

This study still has several methodological limitations, which should be carefully considered when interpreting the results. First, to improve the consistency of citation data and cross-study comparability, only English-language literature was included in this research. This strategy helps ensure data standardization and international readability, but may exclude relevant findings published in regional languages such as Chinese, Japanese and Korean. Although these studies hold certain value in local academic contexts, their dissemination and influence in the global citation network are relatively limited, and thus their impact on the overall research trends is likely to be marginal.

Secondly, Web of Science Core Collection (WOSCC) and Scopus differ in coverage scope, update mechanisms and document type composition (80). WOSCC features a longer time span and a relatively stable citation system, while Scopus offers broader coverage and a higher update frequency. To integrate the advantages of both databases, this study adopted cross-database deduplication and field standardization strategies to reduce systematic bias caused by database heterogeneity. Nevertheless, differences in indexing rules and data structures may still exert a slight impact on institutional affiliation identification and collaboration network construction.

In addition, the standardization of author and institutional names is partially limited by the algorithm accuracy of existing bibliometric tools, which may result in individual matching errors. Nevertheless, given the large sample size and long-time span, such technical biases are not expected to substantially change the overall development trends or core conclusions. It should be emphasized that bibliometric analysis is essentially a macro and retrospective research paradigm. It aims to depict the evolutionary trajectory and knowledge structure of a research field, rather than replacing targeted clinical research or mechanistic investigations. Within this framework, the present study provides a quantitative reference for revealing the research landscape and evolutionary trends in the field of colorectal cancer organoids.

## 5 Conclusion

This study is the first bibliometric analysis of colorectal cancer organoids based on data synthesized from Web of Science Core Collection (WOSCC) and Scopus. Synthesized evidence provides comprehensive insights into emerging research trends and core themes in this field. China and the United States are the primary contributing countries. Hans Clevers is the most influential author. Collaboration among countries and institutions remains to be strengthened. Research on colorectal cancer organoids is maturing gradually with wider application in clinical research in the future. Research hotspots include drug screening and precise modeling of the tumor microenvironment, which hold broad application prospects in precision medicine.

## Data Availability

The original contributions presented in the study are included in the article/**Supplementary Material**. Further inquiries can be directed to the corresponding author.
