# Supplementary figures and images for: Global research trends and hotspots of colorectal cancer organoids: a bibliometric insight and visualization analysis via multiple databases
Source: Front Oncol. 2026 May 18;16:1827951. doi: 10.3389/fonc.2026.1827951 (PMC13222983; doi:10.3389/fonc.2026.1827951)

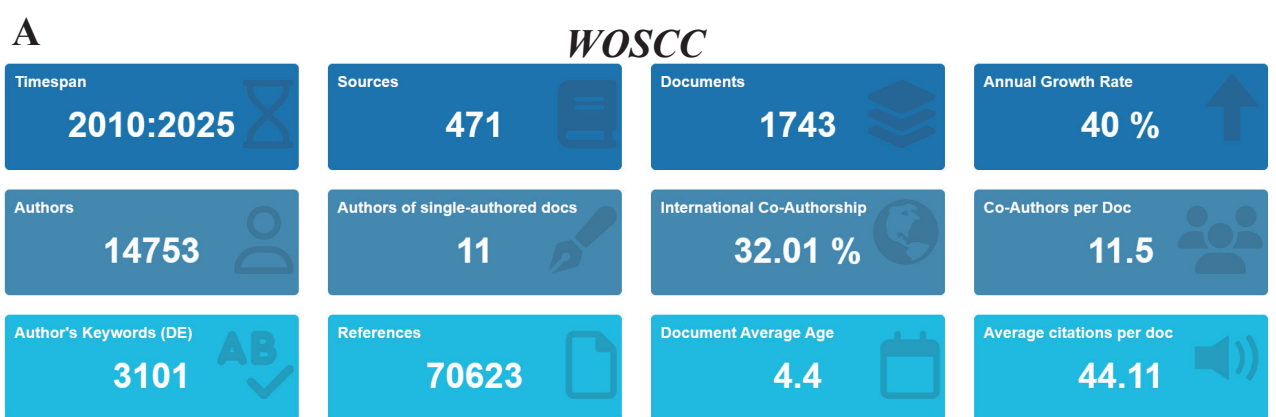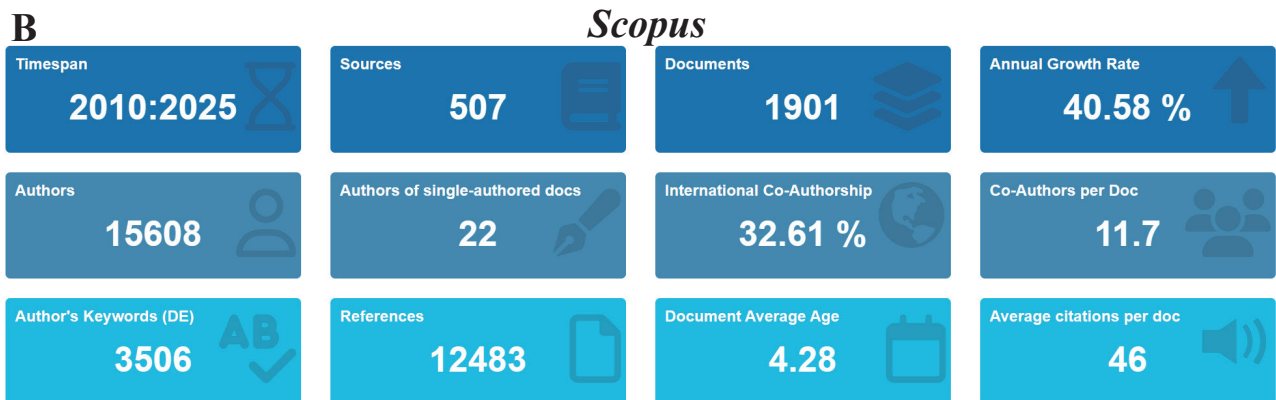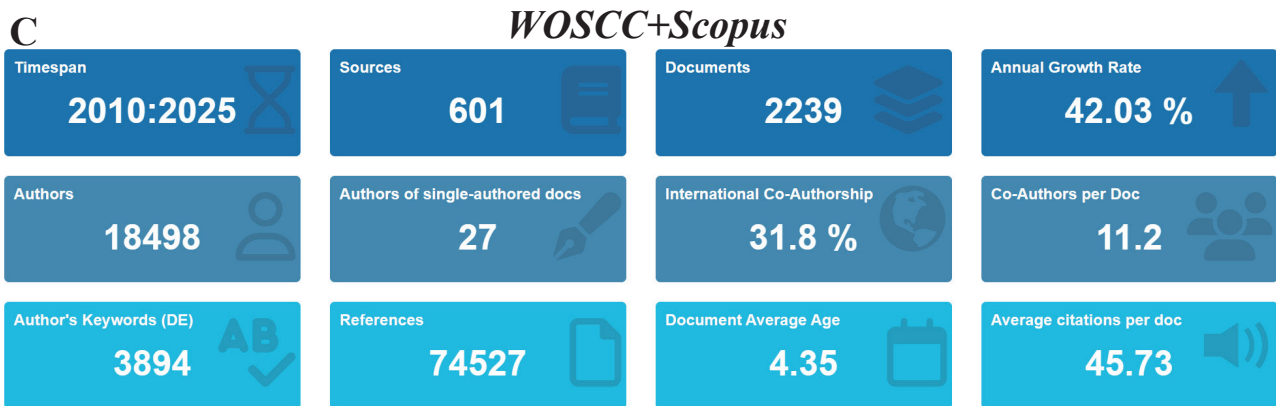

Supplement: Supplementary file 1 [file Image1.pdf]
