# Supplementary material for: Global research trends and hotspots of colorectal cancer organoids: a bibliometric insight and visualization analysis via multiple databases
Source: Front Oncol. 2026 May 18;16:1827951. doi: 10.3389/fonc.2026.1827951 (PMC13222983; doi:10.3389/fonc.2026.1827951)
Supplement: Supplementary file 2 [file Table1.docx]

**Supplementary Table 1.** **Literature search strategies for each database**

| **Database** | **Search Strategy** | **Search Date** | **Filtering Conditions** |
| --- | --- | --- | --- |
| **WOSCC** | TS = ((“Colorectal Tumor* ” OR “Colorectal Neoplasm* ”OR “Colorectal Adenocarcinoma* ”OR “Colorectal Cancer* ” OR “Cancer of the Colorectum ” OR “Colorectal Carcinoma* ” OR “Colon Tumor* ” OR “Colonic Neoplasm* ” OR “Colon Neoplasm* ” OR “Colonic Cancer* ”OR“Colon Cancer* ”OR“Cancer of the Colon ”OR“Colon Adenocarcinoma* ”OR “Rectal Tumor* ”OR “Rectal Neoplasm* ”OR “Rectum Neoplasm* ” OR “Rectal Cancer* ” OR “Rectum Cancer* ” OR “Cancer of the Rectum ” OR “Rectal Adenocarcinoma* ”) AND (“Organoid* ” OR “Tumor Organoid* ” OR “Cancer Organoid* ” OR “Patient-Derived Organoid* ” OR ((“Three-Dimensional ” OR “3D ”) AND (“Organoid* ” OR “Organotypic ”) AND “culture* ”))) | The literature search was conducted on February 09, 2026. | English-language publications from January 1, 2010, to December 31, 2025, including Articles and Review Articles. |
| **Scopus** | ( TITLE-ABS-KEY ( ( “Colorectal Tumor*” OR “Colorectal Neoplasm*” OR “Colorectal Adenocarcinoma*” OR “Colorectal Cancer*” OR “Cancer of the Colorectum” OR “Colorectal Carcinoma*” OR “Colon Tumor*” OR “Colonic Neoplasm*” OR “Colon Neoplasm*” OR “Colonic Cancer*” OR “Colon Cancer*” OR “Cancer of the Colon” OR “Colon Adenocarcinoma*” OR “Rectal Tumor*” OR “Rectal Neoplasm*” OR “Rectum Neoplasm*” OR “Rectal Cancer*” OR “Rectum Cancer*” OR “Cancer of the Rectum” OR “Rectal Adenocarcinoma*” ) ) AND TITLE-ABS-KEY ( ( “Organoid*” OR “Tumor Organoid*” OR “Cancer Organoid*” OR “Patient-Derived Organoid*” OR ( ( “Three-Dimensional” OR “3D” ) AND ( “Organoid*” OR “Organotypic” ) AND “culture*” ) ) ) ) AND PUBYEAR > 2009 AND PUBYEAR < 2026 AND ( EXCLUDE ( DOCTYPE , “no” ) OR EXCLUDE ( DOCTYPE , “ch” ) OR EXCLUDE ( DOCTYPE , “ed” ) OR EXCLUDE ( DOCTYPE , “sh” ) OR EXCLUDE ( DOCTYPE , “er” ) OR EXCLUDE ( DOCTYPE , “le” ) OR EXCLUDE ( DOCTYPE , “cp” ) OR EXCLUDE ( DOCTYPE , “tb” ) OR EXCLUDE ( DOCTYPE , “cr” ) OR EXCLUDE ( DOCTYPE , “dp” ) ) AND ( LIMIT-TO ( LANGUAGE , “English” ) ) | The literature search was conducted on February 09, 2026. | English-language publications from January 1, 2010, to December 31, 2025, including Articles and Review Articles. |

**Supplementary Table 2. Comparison of annual citation count changes across databases**

| **Year** | **WOSCC** | **Scopus** | **WOSCC+Scopus** |
| --- | --- | --- | --- |
| 2010 | 254.50 | 271.00 | 271.00 |
| 2011 | 558.00 | 671.00 | 586.17 |
| 2012 | 435.00 | 416.00 | 307.00 |
| 2013 | 138.38 | 169.50 | 153.06 |
| 2014 | 81.00 | 95.47 | 86.00 |
| 2015 | 262.29 | 277.60 | 245.85 |
| 2016 | 87.61 | 118.76 | 110.15 |
| 2017 | 119.08 | 146.34 | 130.54 |
| 2018 | 96.85 | 105.07 | 107.10 |
| 2019 | 80.11 | 82.47 | 85.24 |
| 2020 | 65.01 | 68.58 | 67.78 |
| 2021 | 32.79 | 35.12 | 33.60 |
| 2022 | 31.07 | 33.29 | 33.17 |
| 2023 | 22.01 | 22.08 | 21.83 |
| 2024 | 10.78 | 15.09 | 14.76 |
| 2025 | 3.07 | 2.93 | 3.26 |

**Supplementary Table 3. Top 10 institutions contributing to publications**

| **Institutions** | **Articles** |
| --- | --- |
| UTRECHT UNIVERSITY | 231 |
| HELMHOLTZ ASSOCIATION | 173 |
| GERMAN CANCER RESEARCH CENTER (DKFZ) | 141 |
| SUN YAT SEN UNIVERSITY | 135 |
| HARVARD UNIVERSITY | 122 |
| FUDAN UNIVERSITY | 111 |
| UNIVERSITY OF CALIFORNIA SYSTEM | 109 |
| UNIVERSITY OF LONDON | 94 |
| UNIVERSITY OF TEXAS SYSTEM | 94 |
| SHANGHAI JIAO TONG UNIVERSITY | 90 |

**Supplementary Table 4. Top 30 Keywords with corresponding frequencies**

| **Keywords** | **Count** |
| --- | --- |
| colorectal cancer | 973 |
| organoid | 563 |
| animals | 359 |
| expression | 347 |
| stem cells | 290 |
| mouse | 226 |
| colon cancer | 223 |
| cells | 212 |
| tumor microenvironment | 209 |
| metabolism | 207 |
| controlled study | 198 |
| colon | 193 |
| genetics | 177 |
| in vitro | 173 |
| pathology | 169 |
| human cell | 165 |
| gene expression | 164 |
| mice | 157 |
| cancer | 149 |
| apoptosis | 144 |

**Supplementary Table 5. Descriptive bibliometric statistics of datasets**

| **Description** | **WOSCC** | **Scopus** | **WOSCC+Scopus** |
| --- | --- | --- | --- |
| Timespan | 2010:2025 | 2010:2025 | 2010:2025 |
| Sources (Journals, Books, etc) | 389 | 417 | 472 |
| Documents | 1450 | 1599 | 1794 |
| Annual Growth Rate % | 44.95 | 46.3 | 47.17 |
| Document Average Age | 4.38 | 4.22 | 4.30 |
| Average citations per doc | 44.11 | 44.95 | 45.44 |
| References | 53220 | 10812 | 55978 |
| Keywords Plus (ID) | 3009 | 12116 | 7773 |
| Author's Keywords (DE) | 2659 | 3012 | 3240 |
| Authors | 13708 | 14561 | 16873 |
| Authors of single-authored docs | 5 | 11 | 13 |
| Single-authored docs | 5 | 12 | 14 |
| Co-Authors per Doc | 12.8 | 12.9 | 12.7 |
| International co-authorships % | 34.69 | 35.08 | 34.67 |
| article | 1450 | 1599 | 1794 |
| review | 0 | 0 | 0 |

**Supplementary Table 6. Publication output and international collaboration patterns of top 10 countries**

| **Country** | **Articles** | **Articles %** | **SCP** | **MCP** | **MCP %** |
| --- | --- | --- | --- | --- | --- |
| CHINA | 412 | 23.0 | 334 | 78 | 18.9 |
| USA | 407 | 22.7 | 279 | 128 | 31.4 |
| GERMANY | 140 | 7.8 | 70 | 70 | 50.0 |
| JAPAN | 135 | 7.5 | 110 | 25 | 18.5 |
| NETHERLANDS | 122 | 6.8 | 67 | 55 | 45.1 |
| UNITED KINGDOM | 95 | 5.3 | 36 | 59 | 62.1 |
| ITALY | 80 | 4.5 | 42 | 38 | 47.5 |
| KOREA | 74 | 4.1 | 66 | 8 | 10.8 |
| AUSTRALIA | 39 | 2.2 | 16 | 23 | 59.0 |
| SPAIN | 35 | 2.0 | 19 | 16 | 45.7 |

**Supplementary Table 7. Top 10 authors by quantitative academic metrics**

| **Author** | **h_index** | **g_index** | **m_index** | **TC** | **NP** | **PY_start** |
| --- | --- | --- | --- | --- | --- | --- |
| CLEVERS HANS | 37 | 50 | 2.313 | 15205 | 50 | 2011 |
| SATO TOSHIRO | 17 | 20 | 1.063 | 7166 | 20 | 2011 |
| YU JUN | 16 | 22 | 2.000 | 1952 | 22 | 2019 |
| GOEL AJAY | 15 | 18 | 1.667 | 698 | 18 | 2018 |
| SANSOM OWEN J. | 15 | 18 | 1.250 | 904 | 18 | 2015 |
| BATLLE EDUARD | 11 | 15 | 0.917 | 3134 | 15 | 2015 |
| FARIN HENNER F. | 11 | 14 | 0.917 | 928 | 14 | 2015 |
| VAN DE WETERING MARC | 11 | 12 | 0.733 | 5265 | 12 | 2012 |
| VOEST EMILE E | 11 | 12 | 0.846 | 2626 | 12 | 2014 |
| KRANENBURG ONNO. | 10 | 24 | 1.250 | 614 | 26 | 2019 |

**Supplementary Table 8. Top 10 journals by quantitative academic metrics**

| **Source** | **h_index** | **g_index** | **m_index** | **TC** | **NP** | **PY_start** |
| --- | --- | --- | --- | --- | --- | --- |
| NATURE COMMUNICATIONS | 29 | 51 | 2.636 | 3502 | 51 | 2016 |
| GASTROENTEROLOGY | 27 | 38 | 1.688 | 5894 | 38 | 2011 |
| NATURE | 23 | 25 | 1.533 | 9868 | 25 | 2012 |
| ONCOGENE | 22 | 39 | 1.571 | 1574 | 47 | 2013 |
| CANCER RESEARCH | 21 | 37 | 1.750 | 1426 | 38 | 2015 |
| SCIENTIFIC REPORTS | 20 | 34 | 1.818 | 1202 | 46 | 2016 |
| CELL REPORTS | 19 | 33 | 1.462 | 1874 | 33 | 2014 |
| CANCERS | 17 | 26 | 2.125 | 821 | 58 | 2019 |
| CELLULAR AND MOLECULAR GASTROENTEROLOGY AND HEPATOLOGY | 17 | 22 | 1.889 | 709 | 22 | 2018 |
| JOURNAL OF EXPERIMENTAL & CLINICAL CANCER RESEARCH | 16 | 28 | 1.6 | 785 | 29 | 2017 |

**Supplementary Table 9. Top 20 cited papers**

| **Paper** | **DOI** | **Global Total Citations** | **Normalized TC** | **IF** |
| --- | --- | --- | --- | --- |
| SATO T, 2011, GASTROENTEROLOGY | 10.1053/j.gastro.2011.07.050 | 3083 | 5.26 | 25 |
| VAN DE WETERING M, 2015, CELL | 10.1016/j.cell.2015.03.053 | 1970 | 7.26 | 42.5 |
| VLACHOGIANNIS G, 2018, SCI. | 10.1126/science.aao2774 | 1616 | 14.99 | 45.7 |
| TAURIELLO DVF, 2018, NATURE | 10.1038/nature25492 | 1510 | 14.01 | 48.4 |
| MATANO M, 2015, NAT MED | 10.1038/nm.3802 | 991 | 3.65 | 49.9 |
| DROST J, 2015, NATURE | 10.1038/nature14415 | 900 | 3.32 | 48.4 |
| MANSOUR AA, 2018, NAT BIOTECHNOL | 10.1038/nbt.4127 | 900 | 8.35 | 41.6 |
| WEISWALD LB, 2015, NEOPLASIA | 10.1016/j.neo.2014.12.004 | 899 | 3.31 | 7.7 |
| CALON A, 2015, NAT GENET | 10.1038/ng.3225 | 890 | 3.28 | 28.9 |
| DIJKSTRA KK, 2018, CELL | 10.1016/j.cell.2018.07.009 | 887 | 8.23 | 42.5 |
| PLEGUEZUELOS-MANZANO C, 2020, NATURE | 10.1038/s41586-020-2080-8 | 881 | 12.90 | 48.4 |
| PAULI C, 2017, CANCER DISCOV | 10.1158/2159-8290.CD-16-1154 | 833 | 6.71 | 33.2 |
| KOO BK, 2012, NATURE | 10.1038/nature11308 | 824 | 2.68 | 48.4 |
| BLOKZIJL F, 2016, NATURE | 10.1038/nature19768 | 747 | 5.81 | 48.4 |
| FUJII M, 2016, CELL STEM CELL | 10.1016/j.stem.2016.04.003 | 717 | 5.57 | 20.4 |
| BEYAZ S, 2016, NATURE | 10.1038/nature17173 | 683 | 5.31 | 48.4 |
| DE SOUSA E MELO F, 2017, NATURE | 10.1038/nature21713 | 638 | 5.14 | 48.4 |
| KIM M, 2019, NAT COMMUN | 10.1038/s41467-019-11867-6 | 612 | 7.43 | 15.7 |
| SHIMOKAWA M, 2017, NATURE | 10.1038/nature22081 | 604 | 4.87 | 48.4 |
| OOFT SN, 2019, SCI TRANSL MED | 10.1126/scitranslmed.aay2574 | 577 | 7.00 | 14.7 |
